# Experimental Study on the Characterization of Orientation of Polyester Short Fibers in Rubber Composites by an X-ray Three-Dimensional Microscope

**DOI:** 10.3390/ma15103726

**Published:** 2022-05-23

**Authors:** Benhui Yu, Jianbin Ren, Kongshuo Wang, Chuansheng Wang, Huiguang Bian

**Affiliations:** 1 College of Electromechanical Engineering, Qingdao University of Science and Technology, Qingdao 266061, China; ybenhui@163.com (B.Y.); renjianbin2021@126.com (J.R.); kongshuo726@163.com (K.W.); 2 Shandong Provincial Key Laboratory of Polymer Material Advanced Manufactorings Technology, Qingdao University of Science and Technology, Qingdao 266061, China

**Keywords:** polyester short fiber orientation, 3Dmed, X-ray three-dimensional microscope, three-dimensional reconstruction, degree of orientation, quantitative characterization

## Abstract

Polyester-short-fiber-reinforced rubber composites have been detected by an X-ray three-dimensional microscope, and then the three-dimensional reconstruction of the image has been carried out to characterize the orientation of polyester short fibers in the composites for the first time. Based on the summary of three traditional methods and mechanisms of characterizing the orientation of polyester short fibers by the numerical parameter method, the direct test method, and the indirect test method, the method and mechanism of the X-ray three-dimensional microscope applied to the orientation characterization of polyester short fibers have been studied. The combination of the center point and threshold segmentation methods has been used to distinguish which fiber section belongs to the same fiber, and the identification of the whole short fiber in different slice images has been realized for the first time. Moreover, Avizo software has been used to realize the three-dimensional reconstruction of a polyester short fiber scanning image. The obtained data have been integrated and the orientation angle and orientation degree have been quantitatively characterized for the first time. This has filled the key technical problem of quantitative characterization of the orientation angle and orientation degree of polyester fibers. The image has been verified by 3Dmed software, and furthermore, the accuracy of the three-dimensional reconstruction results has been verified.

## 1. Introduction

At present, the orientation characterization methods of polyester short fibers mainly include the numerical parameter method, the direct test method, and the indirect test method. The numerical parameter method has been used to describe the orientation of short fibers in a mathematical sense. Direct testing methods include image analysis, ultrasonic technology, and computer simulation. The indirect test method is mainly characterized by the anisotropy of polyester-short-fiber-reinforced rubber composites, including tensile modulus and swelling properties. These methods have some disadvantages, for example, they are unable to quantitatively characterize, have a small analysis area, have high sample preparation requirements, are time consuming, and are unable to quantitatively describe the orientation distribution and orientation degree of short fibers in a statistical sense [1,2,3,4].

The X-ray three-dimensional microscope, which uses X-ray to scan the structure of a sample, provided the two-dimensional sectional image of the internal structure of the sample and then obtained the three-dimensional dialysis image of the sample through image processing to truly show the internal structure of the sample. As an advanced optical imaging technology based on a synchrotron radiation light source, the X-ray three-dimensional microscope is a new three-dimensional perspective microscopic imaging system formed by the combination of traditional CT technology and optical microscopy technology. When the X-ray passed through the sample, a transmission image could be formed on the detector due to the different absorptivity of X-ray by different components in the sample. By rotating the sample, images at different angles could be obtained, and then the image of the three-dimensional structure inside the sample could be reconstructed by image reconstruction technology [5,6,7,8,9,10]. The image of any part in the sample could be obtained through the three-dimensional visualization software, and the part of interest could be rendered and displayed in three dimensions. The X-ray three-dimensional microscope is a new type of high-resolution three-dimensional imaging equipment based on the principle of ray imaging. It can obtain the detailed three-dimensional structure information of the tested object without damaging it. It has been increasingly applied in material science, petroleum geology, advanced manufacturing, and life sciences [11,12,13,14].

**Figure 1:**
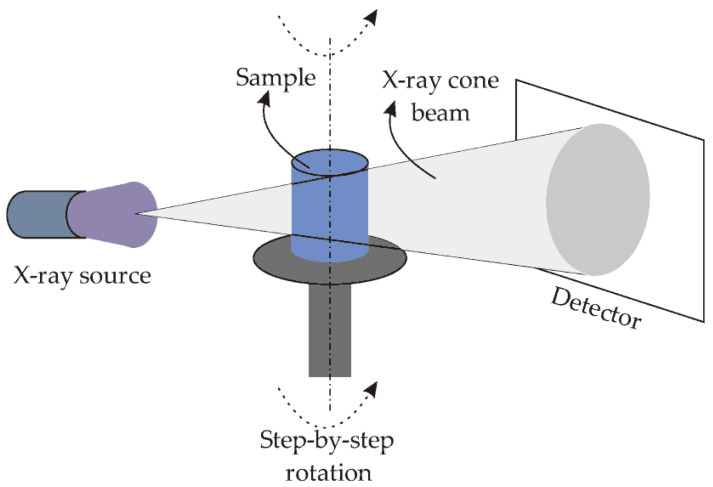
Simplified schematic of the X-ray CT system [26].

As shown in Figure 1, An X-ray three-dimensional microscope (micro-CT) [9,10,15,16,17,18,19,20,21,22,23,24,25,26,27,28,29,30] is mainly composed of a micro-focus ray source system, a precision sample stage system, a high-resolution optocoupler detector, and a flat panel detector system. An X-ray three-dimensional microscope is an instrument developed based on the CT imaging principle. However, the diameter of the polyester fiber is only 20–30 μm, and the difference between the density and the rubber matrix is small, so how to detect polyester short fibers and realize a three-dimensional reconstruction has become a huge problem in polyester fiber detection. The three-dimensional detection of the polyester fiber requires more efficient and advanced instruments and equipment and extremely professional and experienced operators. The cost of professional detection is high. At present, there is no reference on the three-dimensional reconstruction of polyester fibers, and the general conventional tester cannot detect the three-dimensional shape of polyester fibers; in the domestic and international technology, how to distinguish whether the same polyester fiber is in different two-dimensional sections has become the key technical problem of the three-dimensional reconstruction of polyester short fibers. Moreover, the quantitative characterization of the three-dimensional reconstruction of polyester short fibers has not been published in existing articles.

## 2. Experimental

### 2.1. Main Materials

Pretreated polyester short fibers, length: 3 mm, phr(formulation): 1; length diameter ratio: 120, Hebei Baoding synthetic material factory (Hebei, China); carbon black n326, phr: 48, 99.999%; natural rubber, phr(formulation): 100; zinc oxide, phr(formulation): 6, >99%; stearic acid, phr(formulation): 2, standard for GC, >99% (GC); microcrystalline wax, phr(formulation): 25, >99%; Qingdao Railway rubber factory (Qingdao, China).

phr: It means the proportion unit of each component in the formula–mass ratio. For example, assuming that the total mass of the raw materials is m, the total phr is A, and the phr of some kind of raw material named I is A_I_. Then, the mass of the raw material I is m_I_ = m/A × A_I_.

### 2.2. Test Equipment

An Sk-160 two roll open mixer, a product of Dalian Huari Rubber and Plastic Machinery Factory (Dalian China); an X-ray three-dimensional microscope, Tianjin Sanying Precision Instrument Co., Ltd., Tianjin, China.

### 2.3. Preparation of Rubber Compounds

#### Polyester Short Fiber Compounds

**Figure 2:**
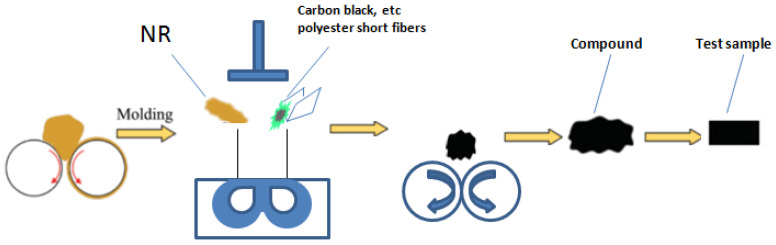
Mixing process.

As shown in Figure 2, the natural rubber was mixed with carbon black and other ingredients by an internal mixer and the rubber was mixed with an open mixer.

The open mixing process was as follows: brought the roll temperature to 40–50 °C; adjusted the roll distance to 2 mm, broke the glue 3 times → adjusted the roll distance to 0.2 mm, made thin pass 3 times → added polyester short fibers along the calendering direction → adjusted the roll distance to 1 mm → adjusted the roll distance to 4 mm and lowered the roll 3 times to obtain polyester-short-fiber-reinforced rubber composites with single orientation along the calendering direction.

Cut the size of the required test sample from the compounds obtained by the roll, i.e., 3 mm × 3 mm × 2.4 mm.

### 2.4. Three-Dimensional Reconstruction Experiments of an X-ray Three-Dimensional Microscope and 3Dmed

#### 2.4.1. Three-Dimensional Reconstructed Images of Avizo Software

Cut a sample of the compounds with a size of 3 mm × 3 mm × 2.4 mm along the short fiber orientation direction (as shown in Figure 2, cut from it). Because the density of the polyester fiber (1.4 g/cm^3^) is similar to that of the rubber matrix (close to 1 g/cm^3^), the absorption rate of X-ray is relatively similar. Therefore, to better detect the morphology of short fibers in rubber, the density gap between them must be increased or the resolution must be sacrificed.

**Figure 3:**
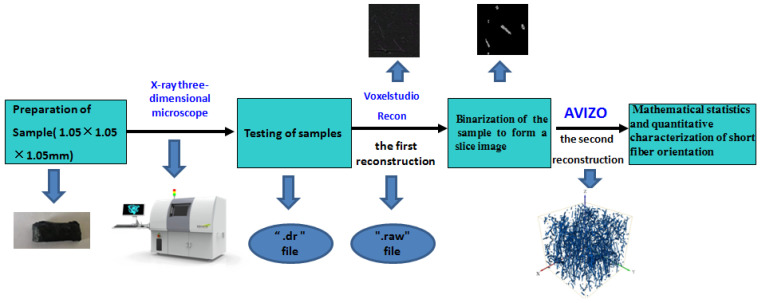
Three-dimensional reconstruction process of polyester fibers.

Figure 3 shows the three-dimensional reconstruction process of polyester fibers in this experiment. The resolution was adjusted to 1.05 micrograms to detect the fibers more clearly. However, adjusting the resolution to 1 microgram meant that the field of view would become smaller. Therefore, only a sample range of 1.05 mm × 1.05 mm × 1.05 mm (resolution 1.05 micrograms; 1000 pixels) could be detected.

**Figure 4:**
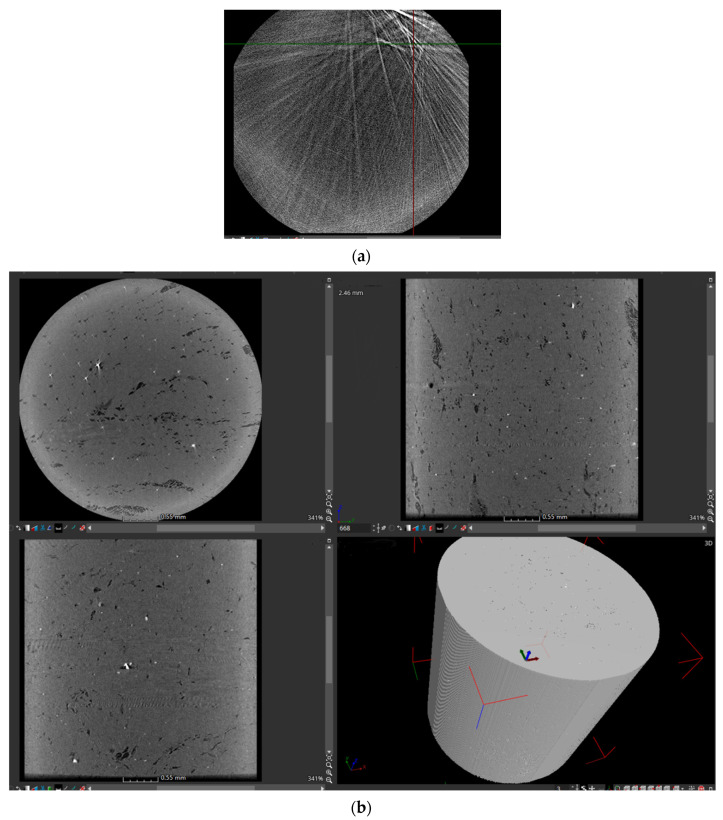
Image of three-dimensional reconstruction with ray artifacts. (**a**) Radial lines, and (**b**) three-dimensional image.

The following were experimental steps of the three-dimensional reconstruction of polyester-short-fiber-reinforced rubber composites detected by the X-ray three-dimensional microscope:

(1) The X-ray three-dimensional microscope scanned the three-dimensional sample and then exported the “.dr” data file.

(2) Special reconstruction software Voxelstudio Recon (Voxelstudio Recon, Tianjin Sanying Precision Instrument Co., Ltd., Tianjin, China) was used for the first reconstruction, and Avizo was used for the second) was used to convert the “.dr” file into a “.raw“ file after debugging and determining that it could overlap. This file had three-dimensional data, which could be imported into Avizo for various calculations and processing and could also be exported to slice images. Figure 4 shows the slice image of a polyester-short-fiber-reinforced rubber composite sample before binarization treatment.

During the first three-dimensional reconstruction of Voxelstudio Recon, as shown in Figure 4, we can see that there are many radial divergent shapes. The reconstructed three-dimensional image in Figure 4 does not appear to correspond to any announced cut from the calendered product because it is not the real image. These radial lines show that the penetration effect of this layer is poor and an overexposure phenomenon occurs. This layer has defects and impurities of a high density, such as metal and silica, which is called the artifact overexposure phenomenon. In this case, the artifact should be filtered. When filtering, the software ImageJ (ImageJ, National Institutes of Health, Bethesda, MD, USA) can be used to identify the corresponding gray levels.

**Figure 5:**
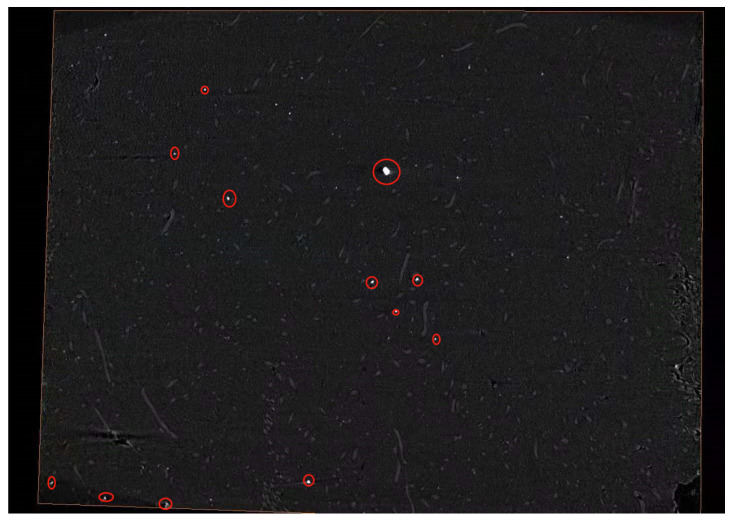
Slice image of a polyester-short-fiber-reinforced rubber composite sample before binarization treatment.

(3) After data processing, the three-dimensional data volume file in “.Raw” format was the three-dimensional data that could be imported into Avizo. Before importing into Avizo, Avizo binarized the images and removed impurity bright spots (as shown in Figure 5, the bright spots in the red circle are impurities of a high density and the long strips are short fibers). Most of the noise of digital image came from the process of image acquisition and transmission, and various factors will affect the work in the process of image acquisition and transmission [31]. Therefore, image filtering was required to remove the impact of these external factors on the images and preserve the detailed feature information of the images as much as possible.

Avizo^®^ Fire software is the advanced three-dimensional visualization and analysis software application for exploring materials science data from tomography, microscopy, MRI, and more techniques. From straightforward visualization and measurement to advanced image processing, quantification, and skeletonization, Avizo Fire provides a comprehensive, multimodality digital lab for advanced two-dimensional and three-dimensional visualization, materials characterization, three-dimensional model generation for FEA, and calculation of physical properties.

**Figure 6:**
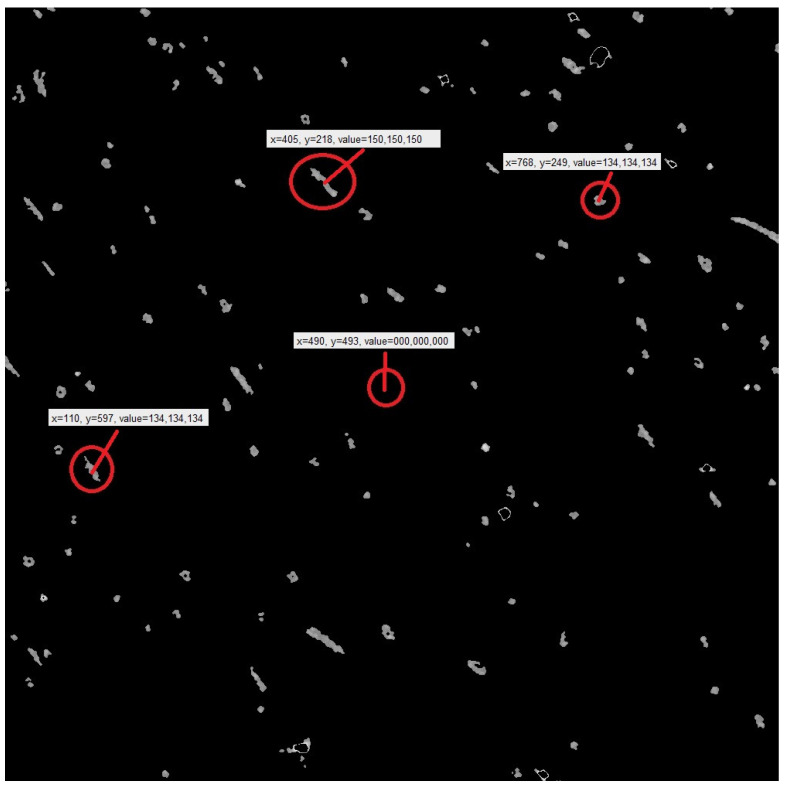
Slice image of a polyester-short-fiber-reinforced rubber composite sample after binarization treatment (image 39).

Then the three-bit data volume was sliced to form a series of slice images (as shown in Figure 6). The series of slice images were imported into Avizo to form a three-dimensional body again. At the same time, the software analysis function of Avizo software was used to determine the orientation angles and other data of the three-dimensional body.

Among them, the data measured by Image J software were as follows: X and Y were the midpoint coordinates of the picture; the value was the gray value. The black-gray value was 0, and the gray of other fibers was greater than 100. After the gray values were measured, the gray of polyester short fibers in different slice directions in the corresponding range could be superimposed to form a three-dimensional reconstructed view.

**Figure 7:**
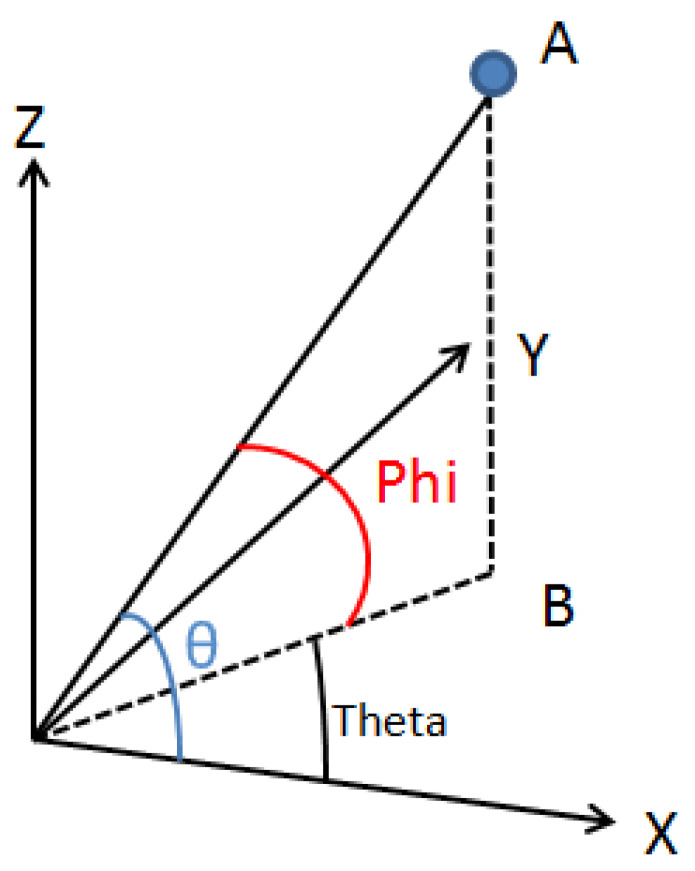
Schematic diagram of Avizo calculating the orientation angle of the polyester-short-fiber-reinforced rubber composite sample after binarization treatment.

In Avizo software, the definition of angle in Avizo software: as shown in Figure 7, the software will export two angles, orientation theta and orientation phi:

Orientation theta: The included angle between the projection of fiber OA on the X–Y loading surface and the *x*-axis, namely ∠XOB.

Orientation phi: The angle between fiber OA and its projection OB on the X–Y section, namely ∠AOB.

Because cosphi=cosθ×costheta, the angle between the fiber and a plane can be obtained by the included angle of the projection on two adjacent planes.

#### 2.4.2. Comparison of Three-Dimensional Reconstructed Images between 3Dmed and Avizo Software

The quality of original data directly affects the accuracy and authenticity of three-dimensional reconstruction results. The images obtained by the X-ray three-dimensional microscope in this test were: (1000 × 1000 × 1000) pixels, 1.05 micrograms per pixel, 1000 tomographic images. The original data of two-dimensional fault sequence of 10 oriented samples, as shown in Figure 5, were selected. The highlighted parts in the fault image are polyester short fibers.

**Figure 8:**
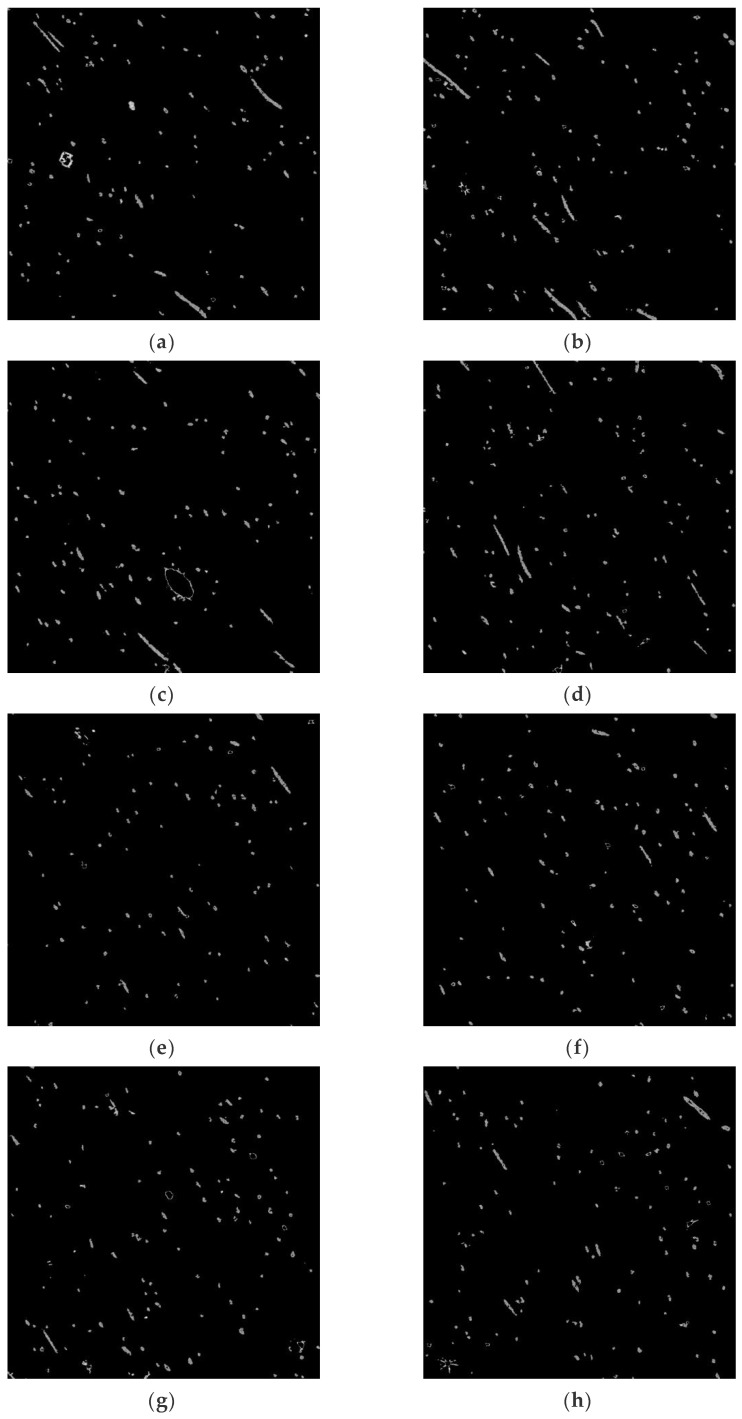

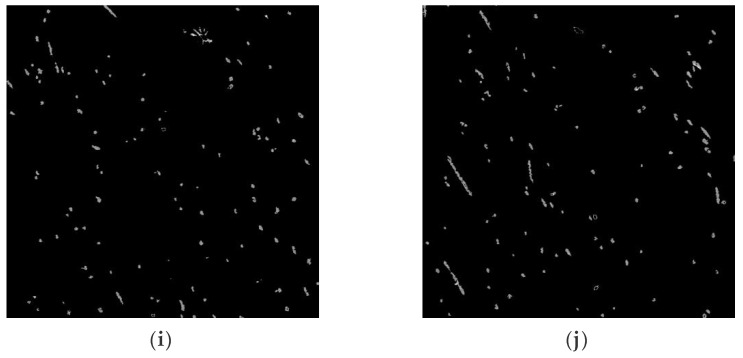
Two-dimensional sectional images of the oriented specimen. (**a**) Image 100; (**b**) Image 200; (**c**) Image 300; (**d**)Image 400; (**e**) Image 500; (**f**) Image 600; (**g**) Image 700; (**h**) Image 800; (**i**) Image 900; (**j**) Image 1000.

Three-dimensional medical image processing and analysis system (3Dmed) was used to read and analyze the two-dimensional slice images in the three-dimensional reconstructed image obtained by the X-ray three-dimensional microscope. Then, data of 1000 slice images, as shown in Figure 8, were loaded in Step 3 to generate a three-dimensional image. The polyester short fiber image could be compared with the three-dimensional structure image formed by Avizo software after 3Dmed three-dimensional reconstruction so as to verify the accuracy of the Avizo three-dimensional reconstruction of polyester short fibers.

## 3. Three-Dimensional Reconstruction and Characterization of X-ray Image

### 3.1. Processing and Analysis of Three-Dimensional Reconstructed Image Based on Avizo

#### Digital Image Processing

Image digitization is the quantitative description of the spatial position and color of the image, which can also be regarded as a process of sampling continuous data. After uniform sampling, the two-dimensional image was discretized into a sheet with (A × B) digital images of samples. The samples in the data image were arranged in A row and B column in a certain order, which were described in the form of a matrix. Digital image processing [32,33] refers to the process of converting image signals into digital signals and processing them by computer, mainly including image transformation, image coding and compression, image enhancement and restoration, and image segmentation.

### 3.2. Identification and Comparison of Polyester Short Fibers

To cooperate with the program of the characterization system, the short fiber images obtained in any way needed to be processed into a gray image to facilitate the identification of polyester short fibers. To quantitatively characterize the orientation and distribution of polyester short fibers, the best pictures to obtain are continuity. Moreover, the thinner the sample, the better the accuracy. After three-dimensional reconstruction by X-ray, the sample was sliced perpendicular to the orientation direction of the short fiber compound body and 1000 pictures in the “*. JPG” format were obtained, as shown in Figure 8, which were imported into Avizo or 3Dmed for image format conversion. When 1000 pictures were imported into Avizo, the pictures in “*. JPG” format were binarized, the pictures were transformed into a gray matrix, and the center point method [1] was used to identify the polyester short fibers in a single image.

Image binarization means to convert the whole image after gray transformation and filter into an image with only two gray value ranges, 0 and 255 [34,35,36,37]. After the binarization operation, the image will become a black-and-white image. The target area and the background area are black-and-white opposite so that they can be completely separated. In the next step, image processing would be more convenient.

**Figure 9:**
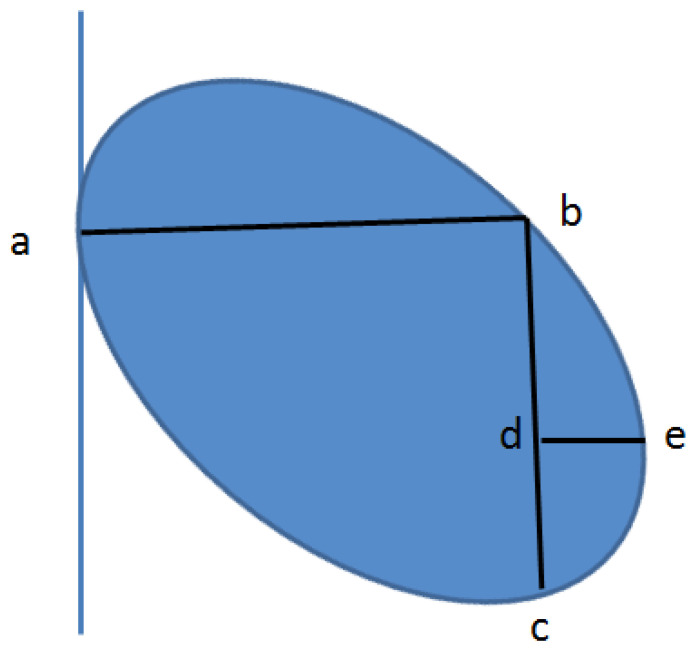
Identification of polyester short fibers by the center point method.

As shown in Figure 9, in the process of obtaining the polyester short fibers’ images, the polyester short fibers would be cut off. All the polyester short fibers in the image sequence were judged and identified. Letter a–e, when a fiber was identified by the center point method, there was a specific distance relationship between a–e, so as to complete the identification of a single fiber [1].

The orientation angles, lengths, and quantity of polyester short fibers in the three-dimensional image restored by the center point were calculated, and the included angle between polyester short fibers and three planes was calculated; after the statistics were completed, the distribution scatter diagram of the orientation angle was drawn.

The three-dimensional drawing was restored from the center point. After the identification of the center point of the polyester short fiber section, the polyester short fibers were reconstructed and then the identified center of the polyester short fibers was projected in a plane.

The obtained slice images were directly superimposed in the thickness direction. By scanning the matrix composed of gray value and point coordinates in each image, the state of polyester short fibers was restored and the real state of polyester short fibers in rubber was obtained.

### 3.3. Identification and Comparison of the Whole Polyester Short Fiber in the Adjacent Slice Images

In the process of calculating short fiber orientation with software Avizo, it was necessary to judge whether the upper and lower fibers of two adjacent slices were the same fiber. The diameter of the short fiber of the sample was calculated at the point on each picture and then the elliptical sphere was calculated at the adjacent points in the two adjacent pictures so as to obtain the angle and length of the fiber. If the adjacent pictures did not coincide, it was considered that the upper and lower fibers were not the same fiber; statistics were made accordingly.

**Figure 10:**
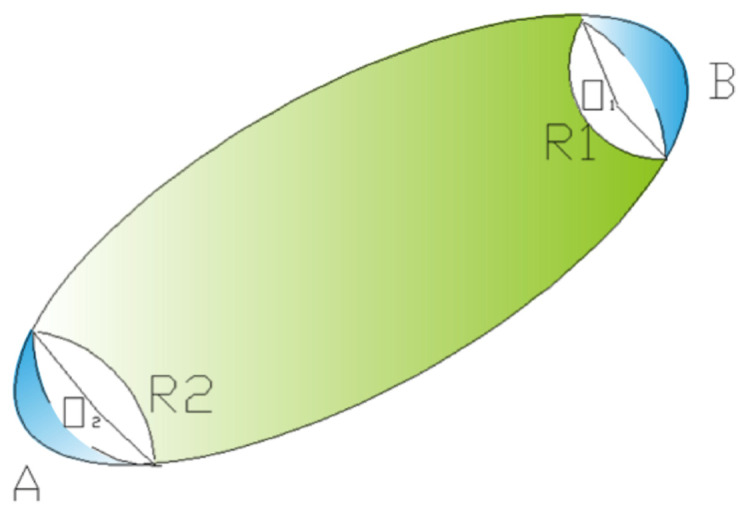
Center point method to identify whether short fibers in different slice sections are the same fiber.

As shown in Figure 10, in the process of obtaining the short fiber images, the short fiber would be cut off. How to judge which two fiber sections belonged to the same whole fiber in adjacent pictures was the focus of this section. The specific methods adopted were as follows:

(1) Find the center point of the fiber section: The center point of each fiber section in each slice image was identified in the previous section, that is, the centroid of the fiber section, such as the radius R1 of the upper slice, the radius R2 of the next slice, and the two centers, $O_{1}$ and $O_{2}$, was calculated.

(2) Judge which fiber the center point in each slice image belongs to i.e., whether the section center point in this slice image belongs to the same fiber as the center point in the adjacent slice image. The judgment was based on projecting the center points of two adjacent slices onto the same plane image to judge the distance between the fiber center points and the threshold value. If it was less than the threshold, i.e., $D_{O_{1}-O_{2}}\leq \left|\;R1-R2\;\right|$, it was considered that the fibers where the two centers were located belonged to the same fiber.

If the distance between a center and all centroids of adjacent slices was greater than the threshold, it was considered that this center was the end point of the fiber, that is, the fiber would not extend into adjacent slices after being sliced.

Specifically, in the two adjacent binarized slices, since the longest distance between the two adjacent slices was 1.05 μm, the distance between the slices in the height direction could be ignored. Therefore, whether the two central points were in the same fiber could be determined only by observing the distance difference in the horizontal direction, $D_{O_{1}-O_{2}}\leq \left|\;R1-R2\;\right|$. That is, when the distance between the fibers of two adjacent slices was not longer than the difference in the radius of adjacent fibers, the adjacent fibers were continuous, which could be considered as two adjacent sections of the same fiber, and so on, until all the fibers were superimposed in the slice thickness direction. On the contrary, if the distance value between the fiber center points of the two adjacent slices was greater than the radius difference value between the upper and lower fibers, the two fibers of the two adjacent slices were discontinuous and one of them was the end face of the other fiber.

(3) Calculate the fiber orientation angle, length, and quantity: The angle between the fiber and three planes was calculated. In calculating the lengths and number of fibers, too-short fibers were treated. If the fiber length was too short or the fiber section was too small, the short fiber was discarded. At the same time, the number of fibers was counted. After the statistics, the distribution histogram of the direction angle and the fiber length would be drawn.

(4) Restore the three-dimensional image by the center point method: After the identification of the center point of the fiber section, the fibers were reconstructed and then the identified fiber center s was projected in a plane to obtain the reconstructed image.

### 3.4. Computer Simulation Results of Polyester Short Fiber Orientation

The orientations of polyester short fibers were simulated and calculated by Avizo software and 3Dmed software. The three parts, identifying the polyester short fibers in each image, identifying the whole polyester short fibers in different images, and restoring the polyester short fibers’ state through image three-dimensional reconstruction, were combined in order. The three-dimensional image obtained by 3Dmed image format conversion was compared with the projection image obtained by the center point to verify the accuracy of polyester short fiber processing. Finally, mathematical statistics were made and the following results could be obtained after calculation.

**Figure 11:**
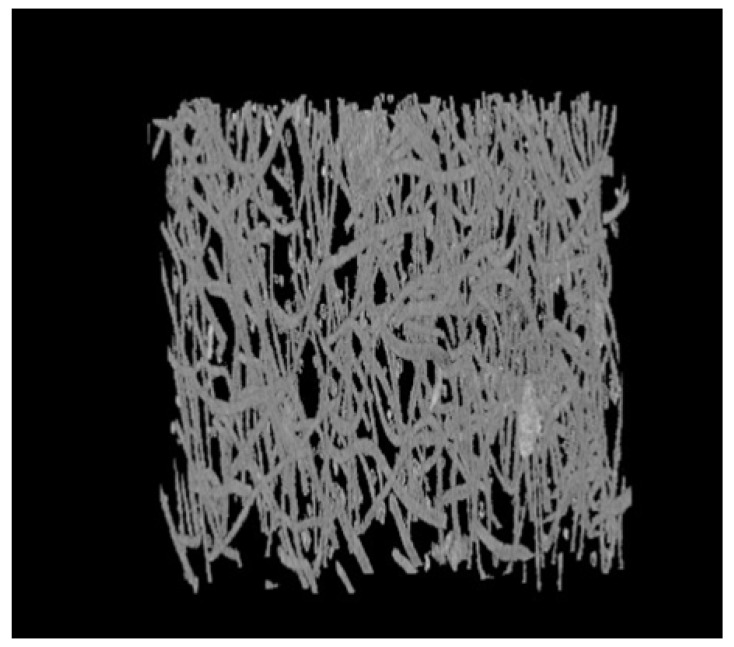
Computer simulation results of 3Dmed-reconstructed polyester short fibers.

**Figure 12:**
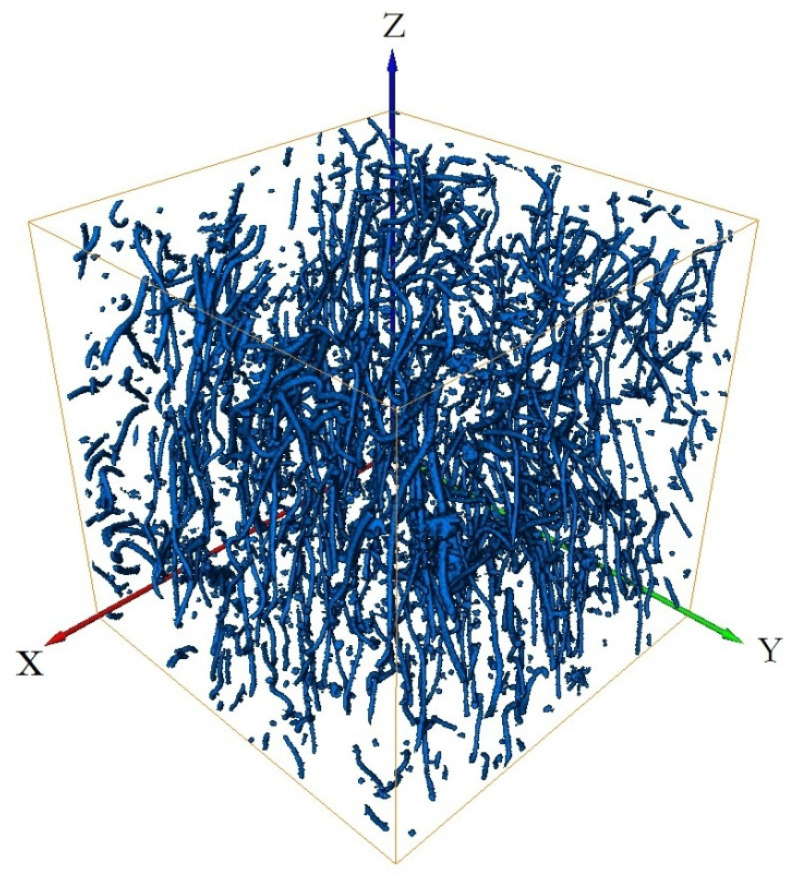
Avizo software reconstructs a polyester short fiber image for the first time.

Figure 11 shows the three-dimensional reconstruction model of the 3Dmed reconstruction. Figure 11 verifies the accuracy of the reconstruction results of Avizo software after X-ray three-dimensional microscope detection in Figure 12. From the three-dimensional reconstruction image, the distribution and orientation of polyester short fibers in rubber can be clearly seen. At the same time, it can also prove that the orientation of polyester short fibers in rubber is single and the degree of orientation is high. It can be seen from Figure 11 and Figure 12 that the reconstructed image has great similarity, so it could be reconstructed and characterized by Avizo software.

Figure 11, Figure 12, Figure 13, Figure 14, Figure 15, Figure 16, Figure 17, Figure 18, Figure 19, Figure 20, Figure 21, Figure 22 and Figure 23 show the reconstructed images of short fibers and the included angle values with three planes XOY, XOZ, and YOZ, as well as the ratio of different lengths and different included angles. The distribution of short fibers in different directions and angles in the sample can be clearly seen from the statistical diagram.

**Figure 13:**
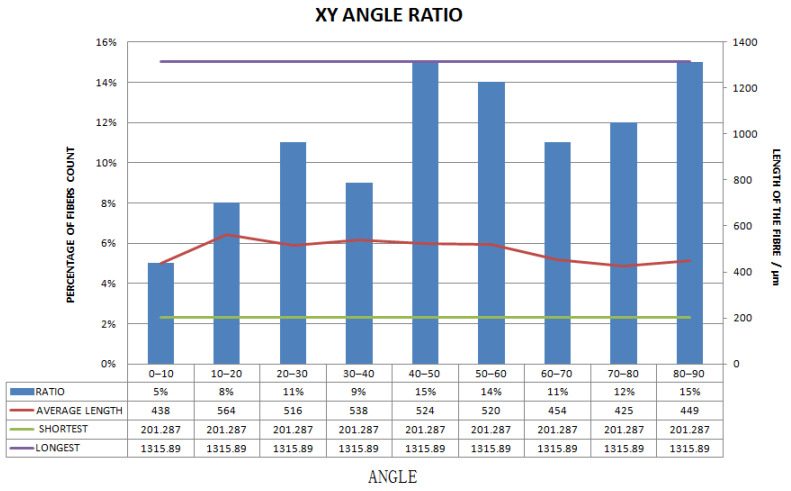
Statistical diagram of the angle between a polyester short fiber reconstructed by Avizo software and the XY direction (less than 200 microns removed).

**Figure 14:**
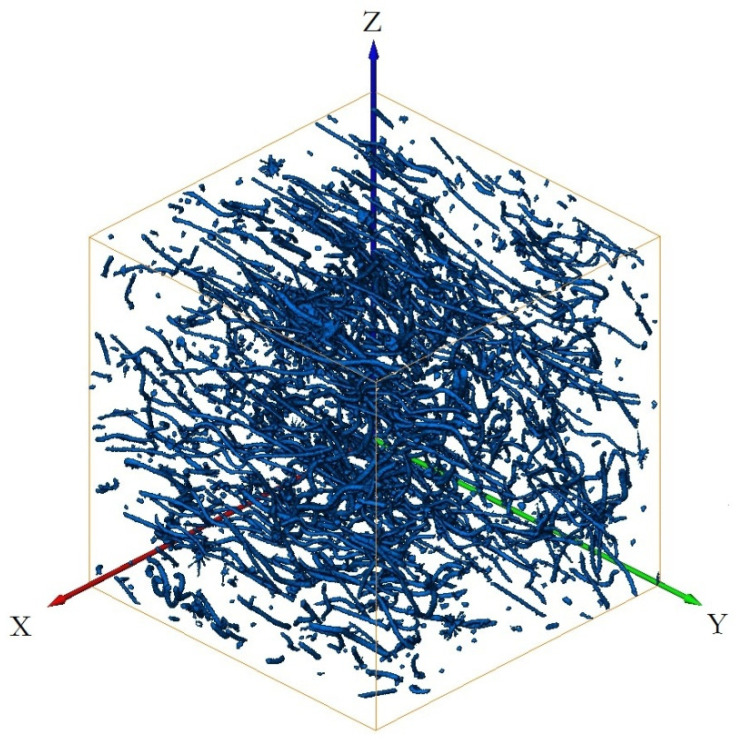
The second reconstruction of polyester short fibers image by Avizo software.

When calculating the angle value and the fiber length of each fiber reconstructed (angle values are between the short fibers and the three planes XOY, XOZ, and YOZ), the three coordinate systems should be transformed. This is because Avizo software could not directly count the other two faces, so it could only indirectly measure the orientation angle through the conversion of the X, Y, and Z coordinate system, that is, fixed a face (XOY face in Figure 12) and converted the X, Y, and Z axes to calculate the included angle between short fibers and fixed face. In Figure 12, red is the *x*-axis, green is the *y*-axis, and blue is the *z*-axis; In Figure 13, red is the *x*-axis, green is the *z*-axis, and blue is the *y*-axis. In Figure 14, red is the *z*-axis, green is the *x*-axis, and blue is the *y*-axis. The coordinate system needed to be reconstructed every time, so it needed to be reconstructed three times to obtain the orientation angle. The accuracy of three-dimensional reconstruction could be verified by comparing the number and length of fibers after transforming the cubic coordinate system each time.

It can be seen from Figure 18, Figure 19 and Figure 20 that the lengths and number of fibers after three reconstructions basically do not change, which proves the accuracy of the reconstructed image after transforming the coordinate systems.

**Figure 15:**
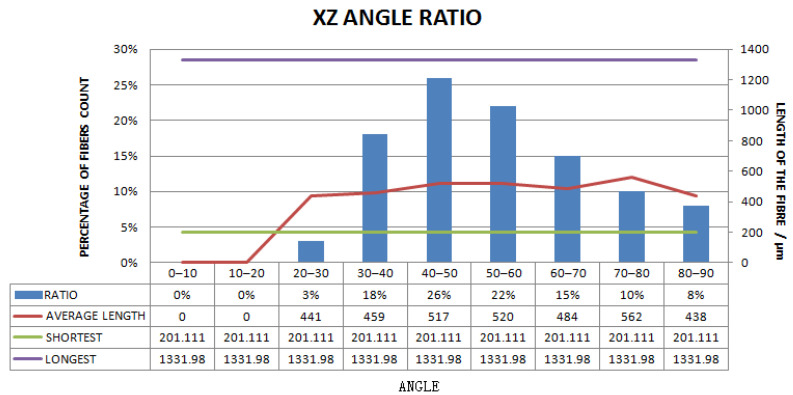
Statistical diagram of the angle between polyester short fibers reconstructed by Avizo software and the XZ direction (less than 200 microns removed).

Figure 13, Figure 15 and Figure 17 are statistical images after discarding fibers’ length less than 200 μm. If the calculated fiber length and number were too huge, the image of this short fiber would be discarded. Here, there was the processing of too-short fibers, that is, if the fiber length was too short, the number of fibers of this length would be deleted. After Avizo statistics was completed, chart data would be formed, as shown in Table 1. According to the chart data, the statistical chart of the orientation angle, the length of each fiber, and the ratio could be drawn. Table 1 shows the maximum cross-sectional area (Area3d), length (Length3d), orientation angle (OrientationPhi), and maximum diameter (EqDiameter) of a single fiber.

Combined with Figure 7 and Table 1, the angle between the fiber and the plane and the proportion of the fiber within a certain orientation angle could be obtained by using the angle relationship between the fiber and the coordinate axis.

The orientation of polyester short fibers was simulated and calculated by Avizo software. The three parts, identifying the polyester short fibers in each image, identifying the whole polyester short fiber in different images, and restoring the state of polyester short fiber through image three-dimensional reconstruction, were combined in order and then mathematical statistics were made. The following results could be obtained after calculation.

**Figure 16:**
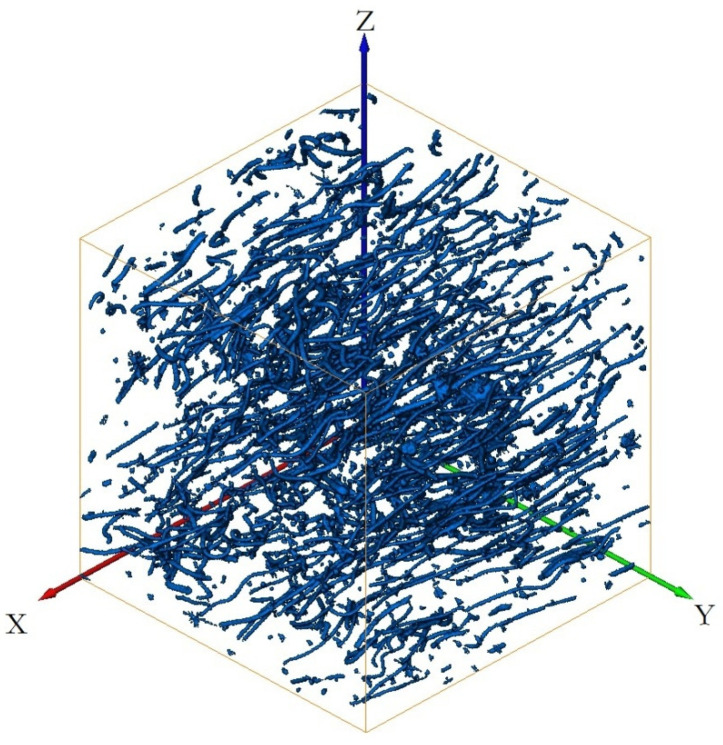
Avizo software reconstructs the polyester short fiber image for the third time.

(1) The three-dimensional reconstructed images by the center point method of the short fiber are shown in Figure 12, Figure 14 and Figure 16.

(2) Polyester short fiber length statistics of 1 mm × 1 mm × 1 mm sample: The polyester short fiber length distribution diagram was created and the average lengths calculated.

As can be seen from Figure 18, Figure 19 and Figure 20, the fiber lengths in the XY direction were 50, 100, 150, 200, 250, 300, 350, 400, 450 and 500, 550, 600, 650, 700, 750, 800, 850, 900, 950, 1000, 1050, 1100, 1150, 1200, 1250, 1300, 1350, 1400 microns and the numbers were 932, 77, 48, 35, 29, 29, 24, 27, 20, 24, 21, 16, 7, 6, 7, 9, 5, 3, 5, 6, 2, 3, 1, 2, 1, 0, 2, 0; 1341 pieces in total.

The fiber lengths in the XZ direction were 50, 100, 150, 200, 250, 300, 350, 400, 450 and 500, 550, 600, 650, 700, 750, 800, 850, 900, 950, 1000, 1050, 1100, 1150, 1200, 1250, 1300, 1350, 1400 mm and the numbers were 931, 78, 48, 35, 30, 30, 23, 23, 26, 20, 23, 15, 6, 7, 7, 8, 6, 4, 4, 5, 3, 4, 1, 1, 1, 1, 1, 1, 0; 1341 pieces in total.

The fiber lengths in the YZ direction were 50, 100, 150, 200, 250, 300, 350, 400, 450 and 500, 550, 600, 650, 700, 750, 800, 850, 900, 950, 1000, 1050, 1100, 1150, 1200, 1250, 1300, 1350, 1400 mm and the numbers were 932, 77, 48, 35, 29, 30, 23, 26, 20, 25, 21, 15, 7, 8, 8, 7, 5, 4, 5, 4, 3, 3, 1, 2, 1, 0, 2, 0; 1341 pieces in total.

From the three reconstruction results after changing the coordinate systems, the average length of fibers and the number of fibers in different length ranges were basically unchanged in each reconstruction, which was well within the error range. Therefore, it can be considered that the image could not be changed after each reconstruction, which could lay a foundation for calculating the included angle between the fiber and each plane in the next step (Step 3).

**Figure 17:**
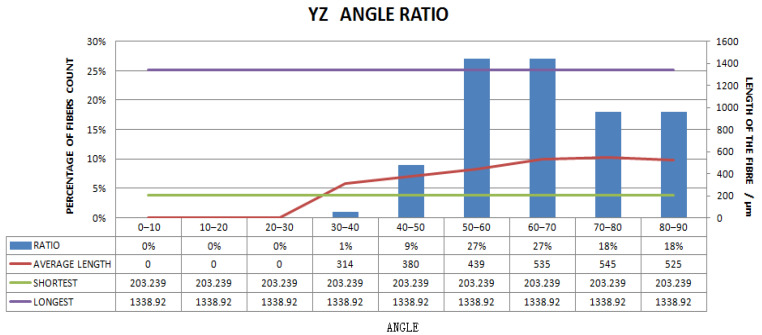
Statistical diagram of the angle between polyester short fibers reconstructed by Avizo software and the YZ direction (less than 200 microns removed).

**Figure 18:**
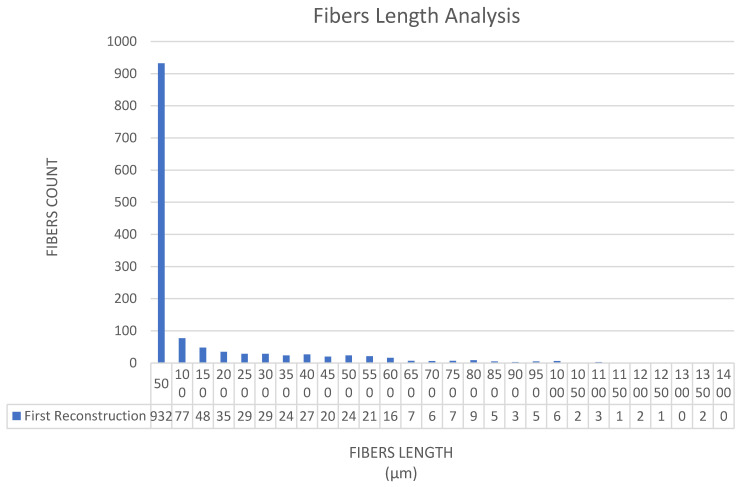
Statistical diagram of polyester short fiber length reconstructed by Avizo software for the first time.

**Figure 19:**
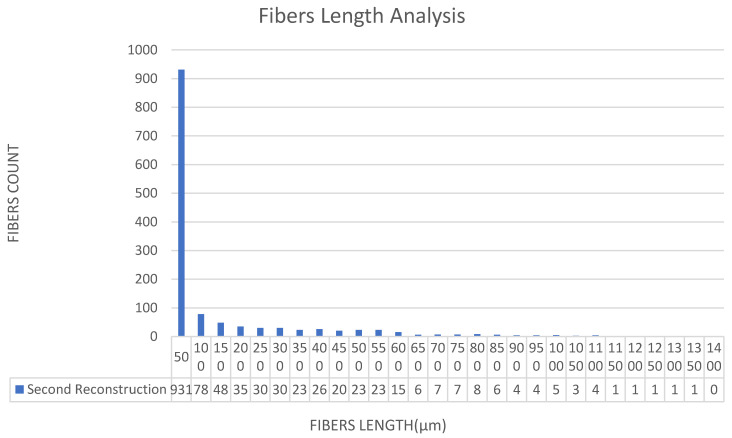
Statistical diagram of the polyester short fiber length reconstructed by Avizo software for the second time.

**Figure 20:**
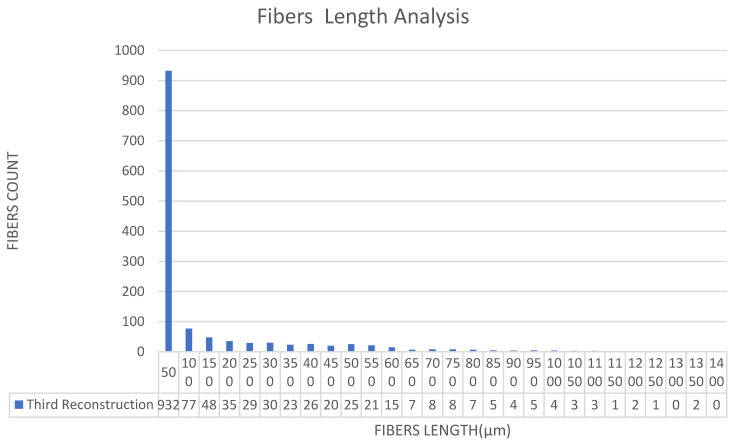
Statistical diagram of the polyester short fiber length reconstructed by Avizo software for the third time.

(3) As can be seen from Figure 13, Figure 15 and Figure 17, the angle with three coordinate planes were included, where X is the width direction of the image, Y is the length direction of the image, Z is the height direction, and the height direction is the slice direction. The orientation of the polyester short fibers could be accurately judged by counting the angle between polyester short fiber and the three coordinate planes.

The number of polyester short fibers with an included angle of 0–10°, 10–20°, 20–30°, 30–40°, 40–50°, 50–60°, 60–70°, 70–80°, and 80–90° with the XY plane accounted for 2%, 4%, 7%, 8%, 9%, 12%, 14%, 20%, and 23%, respectively. The average included angle was 59.42°.

The number of polyester short fibers with an included angle of 0–10°, 10–20°, 20–30°, 30–40°, 40–50°, 50–60°, 60–70°, 70–80°, and 80–90° with the XZ plane accounted for 1%, 2%, 4%, 10%, 13%, 16%, 17%, 18%, and 19%, respectively. The average included angle was 60.44°.

The number of polyester short fibers with an included angle of 0–10°, 10–20°, 20–30°, 30–40°, 40–50°, 50–60°, 60–70°, 70–80°, and 80–90° with the YZ plane was 3%, 7%, 12%, 11%, 12%, 16%, 15%, 12%, and 12%, respectively. The average included angle was 51.29°.

**Figure 21:**
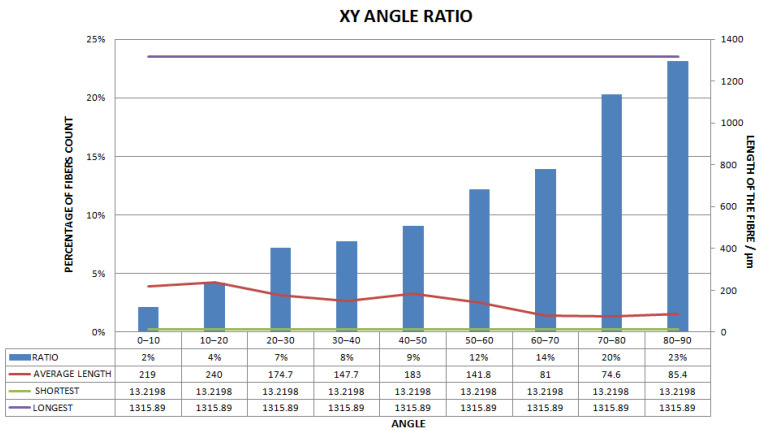
Statistical diagram of the angle between polyester short fibers reconstructed by Avizo software and the XY direction.

**Figure 22:**
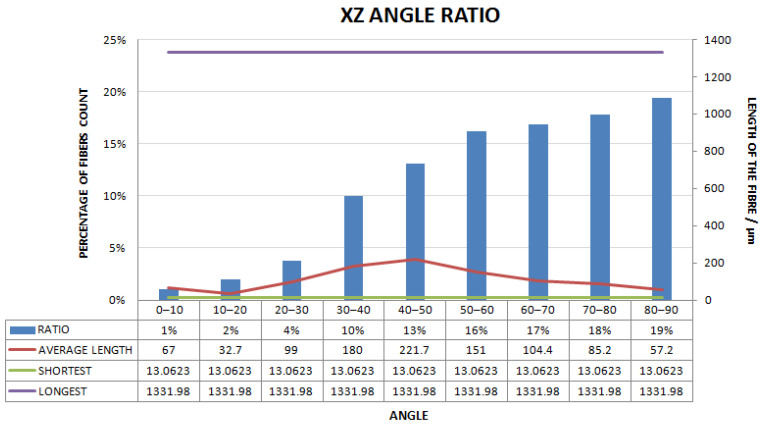
Statistical diagram of the angle between polyester short fibers reconstructed by Avizo software and the XZ direction.

**Figure 23:**
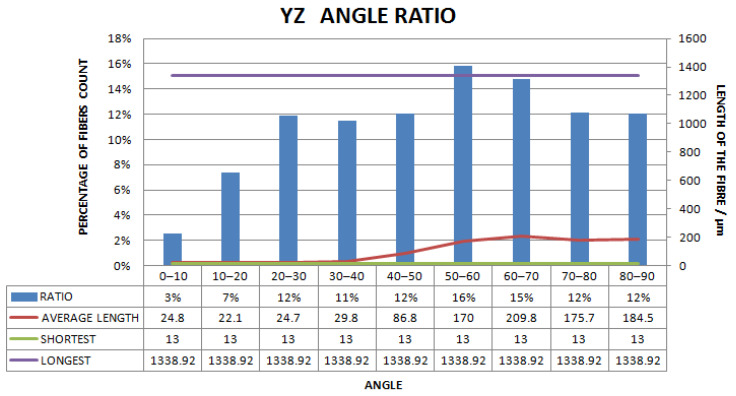
Statistical diagram of the angle between polyester short fibers reconstructed by Avizo software and the YZ direction.

(4) As could be seen from Figure 21, Figure 22 and Figure 23, the fibers less than 200 microns in the image were removed: the length was only 200 microns, and this part might be impurities. Therefore, 409 fibers were left. The results were as follows:

The number ratios of polyester short fibers with an included angle of 0–10°, 10–20°, 20–30°, 30–40°, 40–50°, 50–60°, 60–70°, 70–80°, and 80–90° between fiber and the XY plane were 5%, 8%, 11%, 9%, 15%, 14%, 11%, 12%, and 15%, respectively, and the average angle was 53.68°.

The number ratios of polyester short fibers with an included angle of 0–10°, 10–20°, 20–30°, 30–40°, 40–50°, 50–60°, 60–70°, 70–80°, and 80–90° between the fiber and the XZ plane were 0%, 0%, 3%, 18%, 26%, 22%, 15%, 10%, and 8%, respectively, and the average included angle was 53.12°.

The included angles between the fiber and the YZ plane were 0–10°, 10–20°, 20–30°, 30–40°, 40–50°, 50–60°, 60–70°, 70–80°, and 80–90° and the number ratios of polyester short fibers were 0%, 0%, 0%, 1%, 9%, 27%, 27%, 18%, and 18%, respectively. The average included angle was 64.50°.

(5) The orientation of the fibers calculated by the formula:

(1) $$Average\;value\;of\;included\;angle=\frac{product\;of\;each\;fiber\;angle\;and\;the\;number\;of\;fibers\;under\;this\;angle}{total\;number\;of\;fibers}$$

The average value did not indicate the orientation of the fiber accurately. This is because the longest length of the diagonal of the sample body was so short and so the length of the fiber in the sample was too short (the longest length of the diagonal of the sample body was only 1.4 mm). It was possible to cut a part of the whole fiber in one sample and its orientation became disordered. The ratio of the angle between fiber and the XY plane was more than 70° and the proportion above 70° was 27%. This could be called “the degree of orientation.” It shows the percentage of the total number of fibers on the cone where the angle between the fiber and the XY surface was more than 70° (the fibers of lengths less than 200 microns were removed).

## 4. Conclusions

(1) Based on the obtained results, a new orientation characterization method of polyester short fibers, the X-ray three-dimensional microscope method, has been introduced. The system structure, the system selection, and the three-dimensional reconstruction principle were studied. The oriented polyester-short-fiber-reinforced rubber matrix composites were tested by this instrument, and the computer software Avizo was imported for three-dimensional reconstruction. The correctness of the characterization method has been verified by comparing the 3Dmed image with the Avizo three-dimensional reconstructed image of the polyester short fibers by the center point method. The quantitative characterization of the orientation angle and the orientation angle of polyester short fibers has been realized in three dimensions by using Avizo software to convert the coordinate system, which overcame the defects of the unified characterization method, solved the technical problem that it was difficult to reconstruct polyester fiber in three dimensions, and filled the gap in the characterization of the orientation angle and the orientation angle of polyester fibers in the radial orientation technology of short fibers. Through experiment, the ratio of the angle between fibers and the XY plane was found to be more than 70° and the proportion above 70° was 27% (fibers less than 200 microns in length were removed).

(2) Aiming at the problem of short fiber cutting in the process of obtaining short fiber images, the combination of center point and threshold segmentation was used to judge which fiber section belonged to the same fiber in adjacent pictures and to recognize the whole short fiber dimension of different slice images and three-dimensional reconstruction for different slice images. After the artifact of the image was processed, the accuracy of the reconstruction result was improved. From the reconstruction results, the length of short fibers in each interval of the reconstructed image was basically unchanged. From the results, the accuracy and coincidence rate of the reconstructed image were high. The angle between the short fiber and each surface could be seen in the reconstruction results.

(3) From the combination of center point and threshold segmentation, the angle between the fiber and the plane and the proportion of the fiber within a certain orientation angle could be obtained by using the angle relationship between the fiber and the coordinate axis. In Table 1, Area3d (μm^2^) shows the radius of the fiber at this particular slice location. From the description in Section 3.3 and Figure 10, $D_{O_{1}-O_{2}}\leq \left|\;R1-R2\;\right|$, it was considered that the fibers where the two centers were located belonged to the same whole fiber.

Because the fiber diameter was a fixed value, if the distance between a center and all centroids of adjacent slices was greater than the threshold, it was considered that this center was the end point of the fiber and the fiber would not extend into adjacent slices after being sliced. So at this time, these areas of the two surfaces belonged to different fibers.

The series of slice images were imported into Avizo to form a three-dimensional body again, as shown in Figure 12, Figure 14 and Figure 16. In Figure 12, Figure 14 and Figure 16, at the same time, the software analysis function of Avizo software was used to determine the orientation angles and other data of the three-dimensional body.

The included angle of projection on two adjacent planes (cosθ,costheta): As shown in Figure 7, Avizo’s own calculation program could directly calculate the included angle between each fiber or its projection on any plane and a coordinate axis. To determine the orientation angle, the most important thing was to determine the fiber and take the *z*-axis as a certain angle α, which included the angle of the conical surface, that is, the orientation angle of the fiber in this range.

Because cosphi=cosθ×costheta, the angle between the fiber and a plane could be obtained by the included angle of projection on two adjacent planes. It was assumed that phi + α = 90°, i.e., the included angle phi between the fiber and the XOY surface. Avizo’s own calculation program could be calculated by the formula cosphi=cosθ×costheta. The fiber orientation was obtained by the ratio of the number of fibers within α orientation angle range to the total number of fibers.

## Figures and Tables

**Table 1:** A section of Excel of statistics of fibers in Avizo.

Order	Area3d (μm^2^)	EqDiameter (μm)	OrientationPhi	Length3d (μm)	Index
1	264,931	94.6989	77.2901	1315.89	71
2	121,081	77.9211	42.5806	1309.55	164
3	215,218	92.1428	31.8204	1210.28	41
4	87,404.7	68.9036	58.3198	1196.75	287
5	82,641.6	68.5778	35.6701	1157.19	27
6	53,918.3	57.7063	19.8742	1127.63	48
7	103,179	74.6303	14.9699	1086.5	124
8	69,829	66.1144	45.6686	1080.66	334
9	120,102	75.4554	42.1048	1062.73	201
10	95,093.7	70.3466	81.3404	1040.24	26
11	143,002	80.9693	5.11669	1001	21
12	43,093.3	52.271	29.0356	995.184	6
13	120,898	77.8978	51.2825	993.604	43
14	36,476.2	51.07	60.9781	964.368	188
15	84,127.3	68.3818	23.85	962.495	251
16	47,385.3	58.0941	48.6091	960.1	342
17	54,238.5	57.9373	48.0718	953.391	455
18	34,945.2	51.5429	22.3521	928.395	42
19	49,319.5	58.9736	67.3858	923.234	228
20	63,904.8	63.2888	53.967	922.506	632
21	86,741.7	68.5626	10.6566	910.088	276
22	50,208.5	58.6293	39.7015	903.591	143
23	31,335.2	48.9865	53.2345	887.79	234
24	31,891.1	49.9132	15.6743	882.007	305
25	52,290.5	58.3729	45.5043	877.403	542
26	49,711.7	57.3143	59.4516	839.301	180
27	43,719.8	54.5866	41.3494	827.392	395
28	56,090.6	59.3268	19.8924	812.941	33
29	45,255.6	56.1426	48.8758	811.979	735

## Data Availability

The data presented in this study are available on request from the corresponding author.
